# Dry pleural dissemination of malignancy diagnosed by aggressive thoracoscopy

**DOI:** 10.1002/ccr3.5102

**Published:** 2021-11-19

**Authors:** Akina Nigi, Hirokazu Toyoshima, Motoaki Tanigawa

**Affiliations:** ^1^ Department of Respiratory Medicine Japanese Red Cross Ise Hospital Ise Japan; ^2^ Department of Infectious Diseases Japanese Red Cross Ise Hospital Ise Japan

**Keywords:** adenocarcinoma, dry pleural dissemination, high‐resolution computed tomography, positron emission tomography‐computed tomography, thoracoscopy

## Abstract

Determination of pleural dissemination of lung cancer helps define the treatment strategy. Positron emission tomography‐computed tomography imaging could be false‐negative for dry pleural dissemination of lung cancer. Clinicians should consider preoperative thoracoscopy in affected patients showing limited pleural effusion, interlobar fine granular shadows, and no metastasis on high‐resolution computed tomography.

## Graphical abstract

Aggressive thoracoscopy can help clinicians diagnose dry pleural dissemination, which leads to the appropriate treatment in lung cancer cases with interlobar granular shadows in HRCT even if PET‐CT shows negative uptake in pleural dissemination.

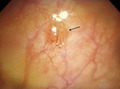

## 1 CLINICAL IMAGE

**FIGURE 1:**
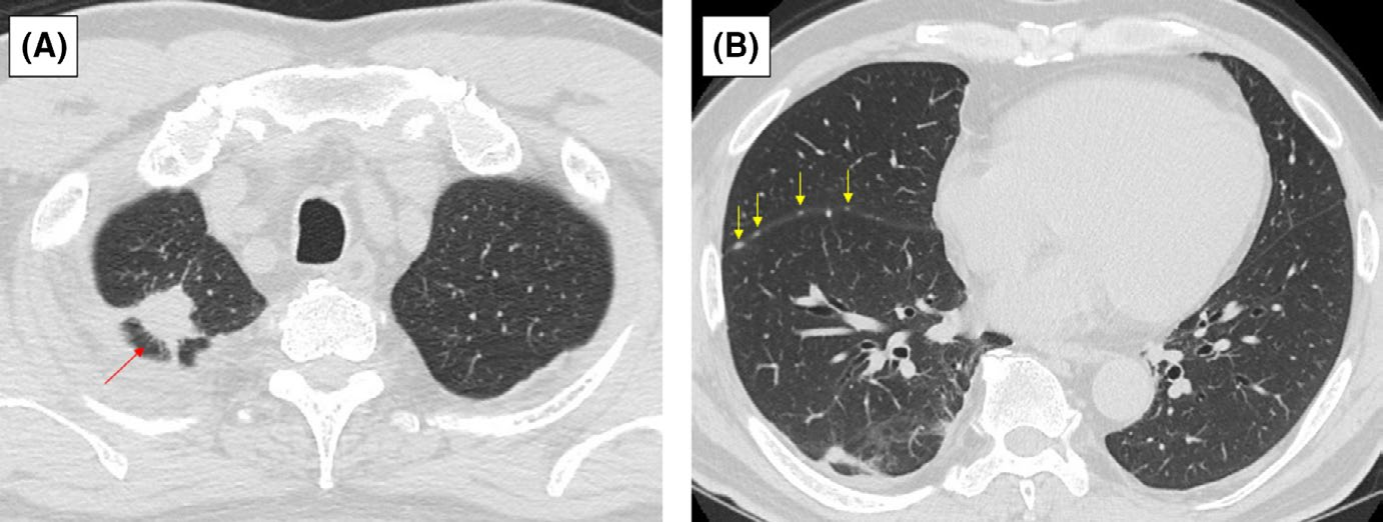
Radiological findings of the patient. High‐resolution computed tomography (HRCT) shows a spiculated 12‐mm nodule (A, red arrow) in the right upper lobe, and fine granular shadows (B, yellow arrows) on the right indicate major fissure. In contrast, right‐sided limited pleural effusion is observed (A, B)

**FIGURE 2:**
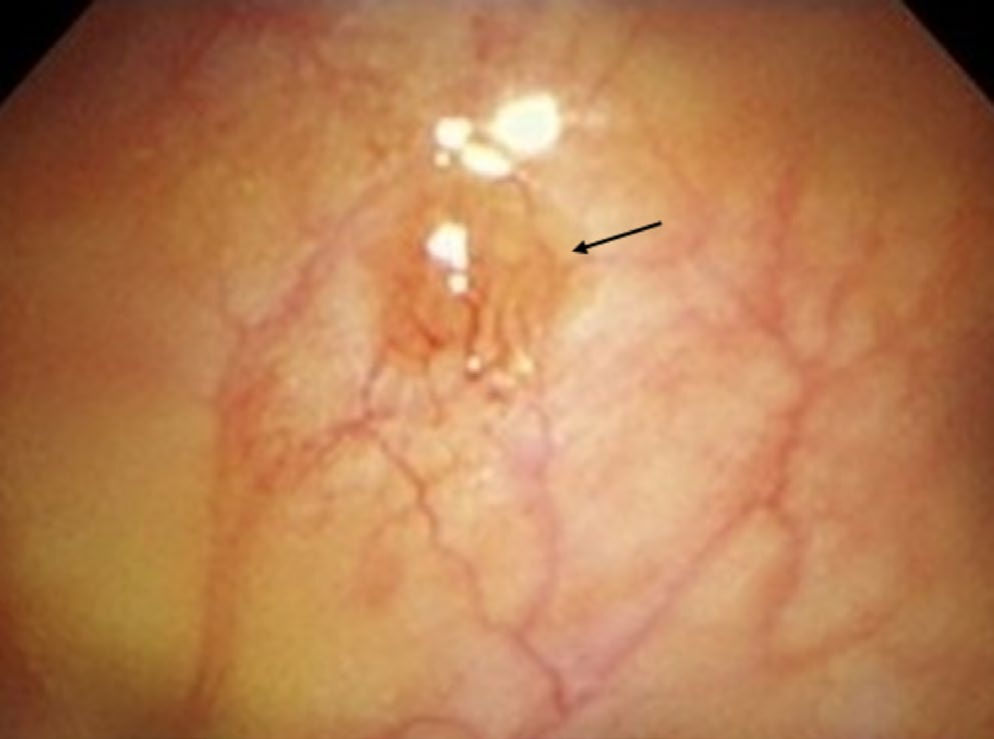
Thoracoscopy reveals jelly‐like lesions with angiodysplasia (black arrow) on the parietal pleura

A 66‐year‐old man was referred to our hospital with suspected lung cancer. Computed tomography (CT) performed with 1.25‐mm slices showed a spiculated 12‐mm nodule in the right upper lobe with limited pleural effusion (Figure 1A, B) and indicated slight interlobar dissemination (Figure 1B) undetected on a 5‐mm slice scan. However, 18F‐fluorodeoxyglucose positron emission tomography‐CT (PET‐CT) demonstrated no uptake, except for that by the tumor. Magnetic resonance imaging showed no brain metastasis. The pleural effusion showed no increase on CT performed 3 weeks later. A local anesthetic thoracoscopy performed for exact staging, revealed sporadic jelly‐like lesions with angiodysplasia (Figure 2) pathologically diagnosed as adenocarcinoma (T1bN0M1a, stage ⅣA) on biopsy.

Local anesthetic thoracoscopy is usually considered in patients with cytology‐negative pleural effusion.^1^ Thoracoscopy in those with limited pleural effusion is challenging due to risk of lung injury. Meanwhile, presence of pleural dissemination without lymph node metastases directly affects the treatment strategy.^2^ We verified the absence of pleural adhesions on ultrasound, which allowed us a blunt and safe approach to the pleural cavity.

An aggressive thoracoscopy may contribute to the diagnosis of dry pleural dissemination of lung malignancy in patients showing no PET‐CT uptake except for that by the primary tumor.

## CONFLICT OF INTEREST

None.

## AUTHOR CONTRIBUTIONS

AN contributed to the clinical management of the patient, was involved in study conception, acquisition and analysis of the data, and drafting of the manuscript. HT was involved in study conception, acquisition and analysis of the data, and drafting of the manuscript. MT was involved in supervision of the drafting of the manuscript and critical revision of the manuscript. All authors reviewed the final draft of the manuscript and approved its submission.

## ETHICAL APPROVAL

This study was approved by the institutional review board and ethics committee of the Japanese Red Cross Ise Hospital (permission number: ER2021‐33).

## CONSENT

Written informed consent was obtained from the patient for the publication of this case report and accompanying images. A copy of the written consent is available for review on request by the Editor‐in‐Chief of this journal.

## Data Availability

The data that support the findings of this study are openly available in [repository name e.g “figshare”] at http://doi.org/10.1002/ccr3.5102, reference number [CCR3‐2021‐09‐1633‐IV.R1].
